# Implications of incorporating morbidity into primary care workload models for NHS funding allocations: a retrospective observational study in England

**DOI:** 10.1136/bmjopen-2025-114094

**Published:** 2026-06-25

**Authors:** Lyvia de Dumast, Patrick Moore, Kym I E Snell, Tom Marshall

**Affiliations:** 1 Department of Applied Health Sciences, University of Birmingham, Birmingham, UK; 2 University of Bristol Medical School, Bristol, UK

**Keywords:** Primary Health Care, Multimorbidity, Health Equity

## Abstract

**Abstract:**

**Background:**

Weighted capitation formulas are used in many countries to allocate primary care funding. Most rely heavily on demographic and area-level factors to weight payments, with limited direct adjustment for morbidity. This may disadvantage practices serving populations with greater morbidity or earlier onset of disease.

**Objectives:**

To assess how well the current National Health Service (NHS) weighting formula reflects morbidity-related workload and how incorporating morbidity would alter national funding allocations.

**Design:**

Retrospective observational study using patient-level electronic health records and national practice-level administrative data. Analyses comprised: (1) practice-level modelling of the NHS practice index (the ratio of formula-adjusted to registered patients); (2) patient-level fixed-effects regression of consultation workload and (3) national simulations applying coefficients from the demographic-only and morbidity-inclusive models to all practices to generate alternative practice weights for comparison.

**Setting:**

Primary care in England (4440 general practices) for practice-level analyses and 627 UK general practices contributing over four million patients to a national electronic health record database for patient-level modelling.

**Primary outcome:**

Annual primary care consultation workload (minutes per patient-year), estimated at patient level and applied in national simulations.

**Secondary outcomes:**

Practice-level predicted workload, morbidity-based practice indices and proportional redistribution under alternative weighting models.

**Results:**

In practice-level analyses of the NHS practice index, the multivariable model explained 77% of variation (R²=0.77). Deprivation, age structure and region accounted for most of this, whereas recorded morbidity contributed relatively little, indicating that the current weighting formula reflects demographic and area characteristics more strongly than morbidity burden. Mean consultation workload was 64.5 min per patient-year. In patient-level models, adding morbidity indicators to the demographic-only specification increased the proportion of variation in workload within practices explained from 16% to 26%. Morbidity was a strong independent predictor of workload and substantially reduced age and sex differences in predicted workload, although socioeconomic differences remained after adjustment. When coefficients from the morbidity-inclusive model were applied nationally to generate alternative practice weights and compared with the demographic-only specification, overall redistribution was modest: practice weights changed by about 2.5% on average. Across practices, 86.7% experienced changes within±5%, while 6.7% gained at least 5% and 6.5% lost at least 5%. Practices serving populations with higher morbidity tended to gain, whereas those serving older populations tended to lose. Practices in more deprived quintiles were significantly less likely to gain and more likely to lose.

**Conclusions:**

The current demographic-based weighting formula captures age and regional variation but only weakly reflects recorded morbidity. Incorporating morbidity improves prediction of workload and produces modest redistribution towards populations with higher disease burden, although deprivation-related differences remain.

**STRENGTHS AND LIMITATIONS OF THIS STUDY**

- Uses complementary national administrative data and patient-level electronic health records to examine demographic and morbidity effects at both patient and practice level.
- Applies morbidity-based workload coefficients nationally across 4440 NHS practices to simulate how incorporating recorded morbidity would alter funding allocations.
- Uses fixed-effects modelling to isolate patient-level associations with workload independently of differences between practices.
- Morbidity was measured using a limited set of 17 recorded long-term conditions and may not fully capture clinical and social complexity, particularly in deprived areas.
- Analyses are based on 2015–2017 data; although consultation volumes may differ in more recent years, the Carr-Hill capitation formula examined has remained structurally unchanged over this period.

## Introduction

 Weighted capitation formulas are widely used to allocate primary care resources according to expected population need. In England, this approach is implemented through the Global Sum—the core capitation payment to general practices—which is allocated using the Carr-Hill formula for practices operating under the General Medical Services (GMS) contract, covering the majority of NHS general practices. The formula adjusts payments according to demographic structure and selected area characteristics, including age, sex, mortality, rurality and residential care. This weighting system therefore plays a central role in shaping how primary care resources are distributed.

In addition to the Global Sum, general practices receive income from performance-related incentives and payments for specific services, including the Quality and Outcomes Framework (QOF) and Enhanced Services. These additional funding streams operate alongside rather than replace capitation payments. As a result, the structure of the Carr-Hill weighting system has important implications for equity in resource allocation across practices serving populations with differing demographic and morbidity profiles.

Although demographic and area-level characteristics function as broad proxies for health need, the current formula does not directly incorporate patient-level clinical morbidity. Evidence shows substantial geographic variation in chronic disease prevalence and associated workload, alongside clear socioeconomic gradients in morbidity burden. Kontopantelis *et al* reported marked spatial variation in chronic morbidity that was not fully reflected in primary care spending.^1^ If morbidity is only imperfectly captured by demographic proxies, practices serving populations with earlier onset or higher cumulative disease burden may receive allocations that do not fully reflect expected workload.

Capitation formulas commonly rely on demographic adjusters such as age and sex. However, models based solely on demographic indicators typically explain only a modest share of variation in utilisation or cost, whereas inclusion of diagnosed morbidity substantially improves predictive performance.^2 3^ In England, concerns have persisted that the current capitation formula does not adequately reflect the distribution of need, particularly in more deprived populations, despite adjustment for demographic and area characteristics. Recent person-based workload modelling has shown that incorporating morbidity indicators increases explanatory power and shifts predicted resource needs toward more deprived populations.^4^ However, less attention has been paid to how explicitly incorporating morbidity into workload prediction would alter funding allocations under the existing capitation framework.

To address this gap, we examine two related questions concerning the workload adjustment within the Global Sum, expressed through the practice index. The practice index—defined as the ratio of formula-adjusted (‘weighted’) patients to the registered list size—determines how demographic and area characteristics translate into capitation payments. First, we assess how effectively the existing demographic specification captures variation in morbidity-related workload across practices. Second, we estimate a morbidity-inclusive workload model and examine how incorporating morbidity alters predicted workload and the distribution of practice-level weights. Finally, by combining national practice-level funding data with patient-level electronic health records (EHRs), we evaluate both the contribution of morbidity to workload prediction and the implications of morbidity-based weighting for resource allocation within the current NHS primary care capitation framework.

## Methods

We conducted a retrospective observational study using routinely collected EHR and administrative data, following the REporting of Studies Conducted using Observational Routinely collected health Data RECORD guideline. The design comprised three related analyses assessing how morbidity affects primary care workload and funding allocations. In this study, workload is measured as annual consultation time per patient, expressed in minutes and weighted by staff role to reflect relative resource intensity. Patient-level workload models were estimated using IQVIA Medical Research Data UK (IMRD-UK), a large anonymised UK primary care EHR database.^5^ Practice-level analyses and simulations were performed using NHS England administrative data. The datasets were analysed independently and were not linked. The study is descriptive and does not attempt to estimate causal effects of morbidity or deprivation on workload or funding.

Throughout, we distinguish between need and workload. ‘Need’ refers to the expected clinical care associated with patient characteristics such as age, sex, morbidity and deprivation. ‘Workload’ represents the recorded clinical activity delivered by practices. Because observed workload reflects both patient characteristics and practice-level factors, it cannot be interpreted as a direct measure of need.

To approximate need more closely, we estimate patient-level fixed-effects models of workload and generate predictions with practice effects set to zero. This isolates the component of workload associated with patient characteristics rather than differences between practices. The resulting predictions approximate the workload expected if practices operated under comparable organisational conditions.

### Data sources

Two datasets were used.

- NHS England practice-level data (2016/17). These data cover all GMS practices. Variables included weighted and registered list size, rurality, the 2015 Index of Multiple Deprivation (IMD), QOF disease registers (from the national pay-for-performance programme in which practices maintain registers of patients diagnosed with specified long-term conditions), the Market Forces Factor (MFF), which adjusts payments for regional variation in staff costs, and health authority identifiers.
- Patient-level IMRD-UK data (2015). Participating practices contribute anonymised EHRs and are broadly representative of the UK population in terms of age, sex and chronic disease prevalence.^6^ Data were extracted using the DExtER tool.^7^ Variables included age, sex, deprivation (Townsend score) and clinically coded long-term conditions diagnoses.^8^

### Overview of analyses

- Analysis 1 (practice-level): regressed the NHS practice index (weighted list size divided by registered list size) on demographic and morbidity characteristics.
- Analysis 2 (patient-level): modelled annual consultation workload to estimate the independent effects of demographic factors and morbidity.
- Analysis 3 (national simulation): applied coefficients from analysis 2 to national practice profiles to simulate morbidity-inclusive practice indices and compare them with demographic-only specifications.

#### Analysis 1: regression of the NHS practice index (NHS England, 4,440 practices)

We analysed 4440 GMS practices using practice-level data on list size, age-sex structure, IMD quintile and QOF registers. The NHS practice index was calculated as weighted list size divided by registered list size and rescaled so that the sample mean equalled 100.^9^ Weighted patient numbers are calculated by NHS England using the Carr-Hill formula, which adjusts for age, sex, morbidity and mortality, residential care, rurality and MFF. We controlled for the main elements of the formula using available demographic and morbidity proxies. Some components could not be replicated directly, but proxy measures were included wherever feasible.

Practices were classified as rural or urban using the rural/urban indicator provided in the NHS England practice-level dataset. Deprivation was measured as a population-weighted average of 2015 IMD scores for Lower Layer Super Output Areas, weighted by the distribution of registered patients.^10^ MFF values were taken from NHS documentation on 2016–17 revenue allocations.^11^ Practices were grouped by health authority.

A multivariable linear regression model was fitted with the NHS practice index as the dependent variable. Covariates were rurality, percentage aged 65 and over, IMD quintile, morbidity burden and MFF. Health authority fixed effects were included and standard errors were clustered by health authority.

Morbidity burden was based on 17 conditions included in the QOF: atrial fibrillation, coronary heart disease, heart failure, hypertension, peripheral arterial disease, stroke and transient ischaemic attack, asthma, chronic obstructive pulmonary disease (COPD), cancer, chronic kidney disease, diabetes, dementia, depression, epilepsy, learning disability, severe mental illness and rheumatoid arthritis. A morbidity index was calculated as the sum of disease-register counts for these conditions divided by practice list size. These registers record the number of patients diagnosed with each specified long-term condition and are used here as proxies for recorded morbidity burden at practice level.

Partial R^2^ values summarised each predictor’s relative contribution using cluster-robust Wald statistics. Full details of model specification and diagnostics are provided in online supplemental appendix 1A,B.

The objective of this model is to examine how observable demographic and morbidity variables relate to variation in the published NHS practice index. The index incorporates demographic and area-based adjustments under the Carr-Hill formula; regressing the index on these variables allows assessment of their relative contribution to variation in the index. The model does not attempt to reconstruct the formula or estimate structural funding parameters.

#### Analysis 2: patient-level modelling and aggregation (IMRD-UK; independent sample)

In analysis 2, patient-level fixed-effects regression was used to control for systematic differences between practices (eg, staffing levels, appointment capacity or coding behaviour). ‘Need-only’ predictions were then obtained by setting practice fixed effects to zero so that predicted workload reflects patient characteristics rather than differences between practices.

#### Outcome

Annual workload (minutes) was calculated as the sum of recorded patient-related activity times, weighted by staff role and relative salary (General Practitioner (GP) 1.0, nurse 0.33, administrator or manager 0.40, other health professional 0.40). The weights were derived from published salary data for each staff group^12^,^15^ to approximate relative resource costs. Similar cost-weighted approaches have been used in previous workload analyses, although the specific role categories and weight values differ. Zero-minute entries were rounded to 30 s and entries over 30 min were truncated.

#### Predictors

Age–sex was defined by 38 groups: 4 infant groups and 34 groups from age 5 to 85 and over. Deprivation was measured with Townsend quintiles, with Q1 representing the least deprived and Q5 the most deprived areas.^16^ Missing deprivation (18%) was multiply imputed using ordinal logistic regression including all analysis variables and the outcome.^17 18^ Full imputation details are in online supplemental appendix 2.

A set of binary morbidity indicators was constructed for 17 long-term conditions included in the QOF framework, based on clinically coded diagnoses. Lifelong conditions were retained indefinitely, while potentially remitting conditions (eg, cancer, asthma, depression) were retained for 5 years following Barnett *et al*.^19^ Additional binary indicators captured recent diagnosis (in the index or prior year) and new registration (in the index or prior year). These variables serve as patient-level measures of recorded morbidity.

#### Model estimation

Fixed-effects linear regression models were fitted with practice identifiers included as fixed effects. Analytical weights equalled person-years, and SEs were clustered at the practice level. Estimates were combined across imputations using Rubin’s rules.^20^

Two models were estimated: (1) a demographic-only model including age-sex, deprivation and new-patient status and (2) a morbidity-inclusive model that additionally included binary indicators for 17 long-term conditions and a recent-diagnosis flag. Because need-only predictions set practice effects to zero, the estimated fixed effects represent differences between observed and predicted workload at the practice level. We report within-practice R² and a practice-level R² from regressing observed practice workload totals on predicted totals using OLS with weights equal to practice person-years, averaged across imputations.

Predicted workloads were normalised to an exposure-weighted mean of 1. Weighted patient values were produced as:


$$w_{i}=\frac{\hat{y_{i}}}{\bar{y}}\times {\text{person-years}}_{i}$$

where y_i_ is the predicted workload for patient *i* and $\bar{y}$ is the national mean predicted workload. Predicted totals were multiplied by exposure for comparison with observed totals.

Practice fixed effects were averaged to summarise unobserved differences. Exposure-weighted means were calculated within deprivation quintiles, trimming the top and bottom 5%. Deprivation quintiles were defined from the modal Townsend score. A sensitivity analysis used only observed deprivation data.

Calibration was summarised by the slope and intercept from the practice-level regression. As a robustness check, models were re-estimated using consultation counts as the outcome with fixed-effects negative binomial regression (online supplemental appendix 9).

#### Analysis 3: practice-level simulation using IMRD-UK coefficients

This analysis used the same 4440 NHS practices as in analysis 1. Indicators for new patients and recent diagnoses were not available in the national administrative dataset; therefore, the morbidity specification applied in this simulation excluded those components of the patient-level model.

We applied the patient-level coefficients from analysis 2 to each practice’s demographic and morbidity profile to generate predicted workload per registered patient. These predictions were aggregated to practice level, scaled relative to the national mean, and expressed as a practice index (mean=100). Differences between the demographic-only and morbidity-inclusive indices summarise the implied redistribution under a morbidity-based approach.

Redistribution from introducing morbidity adjustment was summarised by the proportional change:


$$\Delta =\frac{I_{\text{morbidity}}}{I_{\text{demographic}}}-1$$

We summarised Δ using the median, IQR, 10th and 90th percentiles, the mean absolute proportional change (MAPC) and the Wald share, which represents the proportion of total weighted patients that would need to be redistributed between practices for morbidity-based weights to replace demographic-only weights, assuming national totals remain constant. The formal definition is provided in online supplemental appendix 10.

Symmetry of Δ was assessed using visual checks and summary statistics. Subgroup summaries used IMD quintile and region. Determinants of redistribution were examined by regressing Δ on practice characteristics with region fixed effects and patient weights. Binary outcomes were modelled using linear probability models with robust standard errors clustered by health authority.

## Results

Descriptive characteristics of the practice-level and patient-level datasets are shown in table 1. The NHS administrative dataset comprised 4440 practices covering 33.3 million registered patients, while the IMRD-UK dataset included over four million patients from 627 contributing general practices in 2015. Key demographic, socioeconomic and morbidity characteristics are summarised to provide context for the regression analyses.

**Table 1:** Descriptive characteristics of NHS and IMRD datasets

Characteristic	NHS dataset (practice-level)	IMRD dataset
Total registered patients	33.3 million	4.5 million
Mean list size (SD)	7503 (4301)	9240 (4296)
Mean age (SD)	–	42.7 (23.9)
% female	49.9	53.6
% aged ≥65	17.2	20.7
% IMD Q1 (least deprived)	20.1	22.3
% IMD Q5 (most deprived)	19.9	14.8
Mean morbidity index (SD)	0.53 (0.12)	0.65 (0.13)
Mean annual workload, minutes (SD)	–	64.5 (84.1)
Market forces factor (SD)	1.17 (0.10)	–

Morbidity index=total QOF disease register counts (across 17 conditions) divided by practice registered list size.

IMD, Index of Multiple Deprivation; IMRD, IQVIA Medical Research Data; NHS, National Health Service; QOF, Quality and Outcomes Framework.

Findings from the IMRD patient-level models and the NHS practice-level analyses were consistent and revealed three main patterns. First, the current NHS demographic formula is shaped predominantly by age, deprivation and region, with morbidity contributing relatively little to practice-level variation. Second, morbidity is a strong predictor of workload at patient level, substantially improving explained variation compared with demographic factors alone. Third, applying morbidity-inclusive coefficients nationally results in only modest overall redistribution, but systematically increases weights for practices with higher morbidity and lower weights for those serving older or less deprived populations. The following sections present these findings in detail.

### Analysis 1: regional variation in practice indices

Of 7763 general practices in England in 2016/17, 4440 were included after excluding practices with incomplete contracts, atypical characteristics, or missing or implausible data. Summary characteristics are shown in online supplemental appendix 3. The practice index (ratio of weighted to registered patients where weights reflect the NHS funding formula, rescaled to a mean of 100) ranged from 56.1 to 148.8 (SD 9.9).

The regression model explained a large proportion of variation in the practice index (R² 0.769). Urban practices had lower indices than rural practices (−6.1, 95% CI −6.8 to −5.3). Each one percentage-point increase in the proportion of patients aged 65 years and over was associated with a 1.19-point higher index (95% CI 1.14 to 1.25). Practices in more deprived areas also had higher indices: the most deprived quintile was 19.6 points above the least deprived (95% CI 17.7 to 21.4). Higher morbidity burden (9.4, 95% CI 4.4 to 14.5) and higher MFF (25.9, 95% CI 17.5 to 34.3) were also associated with higher indices.

Partial R² values showed that deprivation, age and geography each accounted for about 19% of explained variance, followed by rurality (18%), MFF (15%) and morbidity (11%) (online supplemental appendix 4). Morbidity therefore contributed relatively little to variation in the practice index compared with demographic and area-based factors.

These findings suggest that the current weighting formula reflects socioeconomic and demographic characteristics more strongly than morbidity burden. We therefore next examined patient-level workload to assess how much morbidity contributes independently of demographic factors.

### Analysis 2: patient-level modelling (IMRD-UK)

The IMRD-UK dataset for 2015 included over four million patients with linked demographic and practice information. Mean workload was 64.5 min per patient-year (SD 84.1), with substantial variation between practices.

#### Model performance

The demographic-only model explained 16.1% of within-practice variance. Adding QOF morbidity indicators increased this to 26.3%. At practice level, predicted totals closely matched observed totals (R² 0.80 in both models).

#### Patient-level predictors

Because the models include practice fixed effects, coefficients represent within-practice associations and therefore reflect differences in workload attributable to patient characteristics rather than practice-level factors such as staffing or coding behaviour. The resulting ‘need-only’ estimates approximate how workload would vary if practices operated with similar supply capacity. Key coefficients are in table 2; the full set is in online supplemental appendix 5.

**Table 2:** Key coefficients from the patient-level fixed-effects regression

Covariate	Coefficient	95% CI
Deprivation (ref:Q1)		
Q2	1.11	0.83 to 1.40
Q3	2.96	2.62 to 3.29
Q4	5.60	5.23 to 5.96
Q5	8.33	7.90 to 8.77
New patient	7.71	7.10 to 8.32
Recent diagnosis	16.5	15.80 to 17.24
Morbidity indicators	See figure 2	

Workload was positively associated with age, female sex, deprivation and new patient status. In the demographic-only model, patients in the most deprived quintile (Q5) required 13.7 more minutes per year than those in the least deprived (Q1). In the morbidity-inclusive model this difference fell to 8.3 min. New patient status was strongly associated with workload: +8.1 min (95% CI 7.5 to 8.8) in the demographic-only model and +7.7 min (95% CI 7.1 to 8.3) in the morbidity-inclusive model.

**Figure 1:**
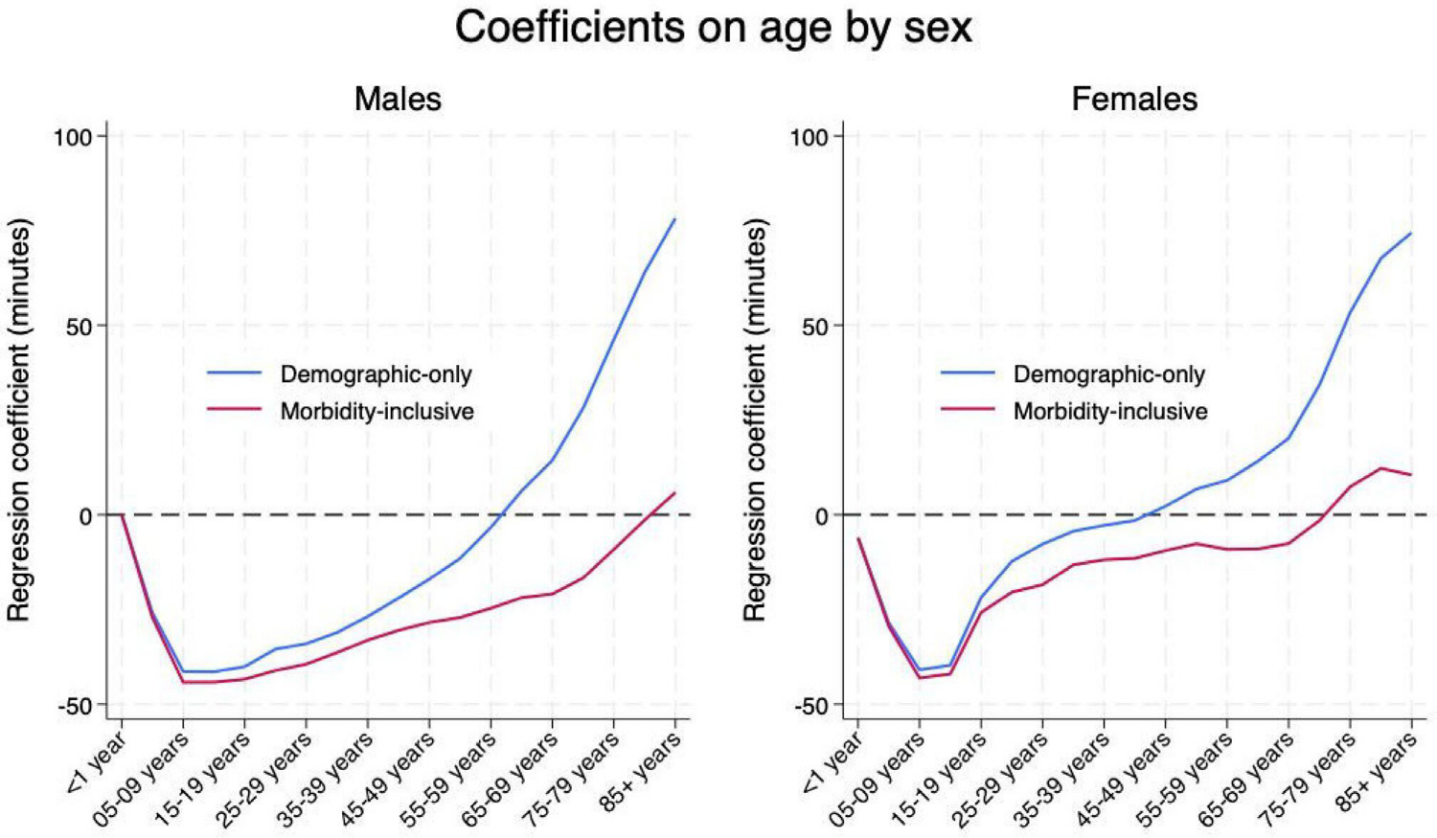
Estimated age–sex coefficients (minutes per patient-year) from demographic-only and morbidity-inclusive workload models.

Workload rose steeply at older ages. From around age 5, females had higher workloads than males, with the gap largest in early adulthood and narrowing at older ages. Age–sex effects were attenuated once morbidity was included (figure 1). Coefficients are expressed relative to males aged under 1 year. Overall, coefficients were smaller in the morbidity-inclusive model, indicating that much of the age–sex gradient was explained by morbidity.

**Figure 2:**
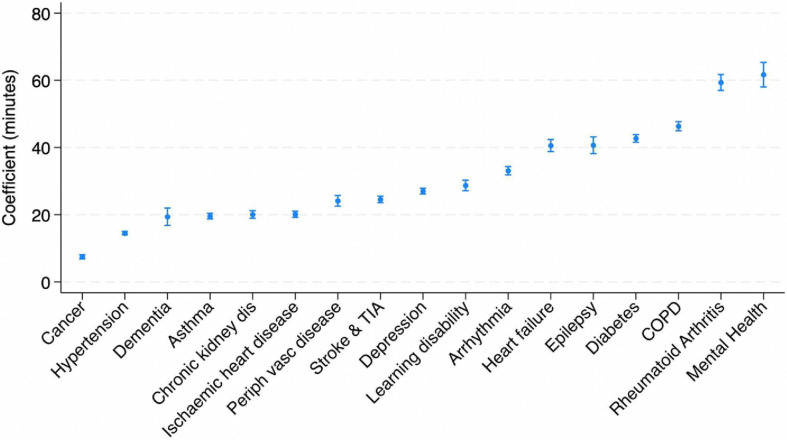
Estimated coefficients for long-term conditions (minutes per patient-year). COPD, chronic obstructive pulmonary disease; TIA, transient ischaemic attack.

Morbidity was a strong predictor. All coefficients were positive and significant, with large associations for diabetes (+42.7 min, 95% CI 41.6 to 43.8), COPD (+46.3 min, 95% CI 45.0 to 47.7) and dementia (+19.5 min, 95% CI 16.9 to 22.0). A recent diagnosis was associated with an additional 16.5 min (95% CI 15.8 to 17.2). Figure 2 shows estimated coefficients and 95% CIs.

#### Practice fixed-effects

**Figure 3:**
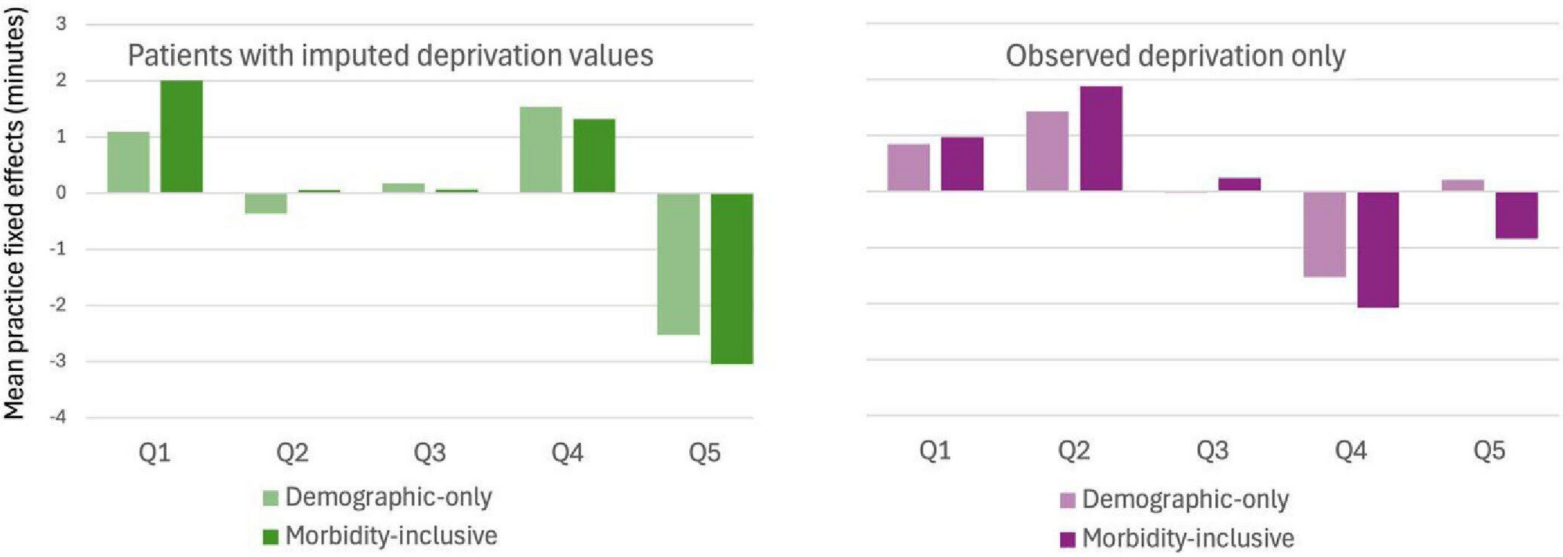
Mean practice fixed-effects by deprivation quintile. Bars show exposure-weighted means, trimmed at the 5th and 95th percentiles. The left panel includes imputed deprivation values; the right panel uses observed data only. Positive values indicate higher-than-predicted workload; negative values indicate lower-than-predicted workload.

Practice fixed effects varied by deprivation quintile (figure 3). With multiply imputed deprivation, the pattern was weak and non-linear: higher-than-predicted workload in quintiles 1 and 4 and lower in quintile 5. Using observed deprivation data only produced a clearer gradient: practices in Q4 and Q5 delivered less workload than predicted, while Q1 and Q2 delivered more. Results were similar using alternative summary measures (online supplemental appendix 6). Including morbidity strengthened these deprivation-related differences. If unmeasured morbidity were driving the pattern, differences would be expected to narrow; instead, they widened, suggesting supply-side constraints in more deprived areas.

Robustness checks (online supplemental appendix 6) gave consistent results.

##### Patient weightings

Under the demographic-only model, mean weights rose from 0.95 in Q1 (least-deprived) to 1.07 in Q5 (most deprived), a difference of +0.12. Under the morbidity-inclusive model, the difference widened slightly to +0.14.

### Stratification by age and deprivation

**Figure 4:**
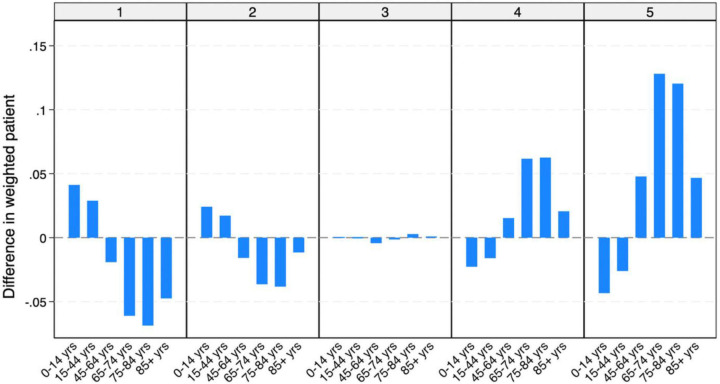
Change versus demographic-only model by age band and deprivation quintile.

Figure 4 shows that deprivation differences were largest at older ages. In Q4 and Q5, weights increased for patients aged 65 and over and decreased for children and younger adults. In Q1 and Q2, weights increased slightly for children and declined for older adults. Quintile 3 weights were close to zero across all ages.

### Sensitivity analysis

Re-estimating the models using consultation rates produced similar results: the morbidity-inclusive model again outperformed the baseline and the same age, deprivation and fixed-effect patterns were observed. This indicates that findings do not depend on the workload metric.

### Practice indices within IMRD practices

We next examined how patient-level predictions from analysis 2 translated into practice-level patterns within the IMRD sample. This intermediate step assessed whether the redistribution implied by the morbidity-inclusive model was already visible within the IMRD practices before extending the coefficients to all NHS practices in Analysis 3.

The morbidity-based practice indices showed a modest redistribution relative to the demographic specification (MAPC 2.5%). Practices with higher morbidity tended to gain relative share, while those with older or less deprived populations tended to lose. A clear deprivation gradient remained, indicating that recorded morbidity does not fully capture deprivation-related workload. These patterns were also seen in the national administrative data presented in analysis 3, although the IMRD sample showed slightly more compressed gains and losses (full redistribution statistics are provided in online supplemental appendix 7).

### Analysis 3: NHS practice-level simulation

To test whether the patterns observed in IMRD generalised to all English practices, we conducted national simulations using practice-level data for 2016/17. We constructed a demographic-only baseline practice index and a morbidity-inclusive index by applying coefficients from the IMRD models to all 4440 GMS practices. Each index was rescaled so that the patient-weighted national mean equalled 100. The proportional change (Δ) compared the morbidity-inclusive and demographic-only practice indices and had a patient-weighted mean close to zero. These simulations illustrate how practice funding shares would change if weights were based on predicted workload rather than the demographic-only formula.

The distribution of Δ was centred close to zero and approximately symmetric, with nearly identical mean and median values (−0.0005 and −0.0010) and low skewness (0.29). There was no evidence that the median differed from zero, supporting the use of both mean/SD and median/IQR summaries (online supplemental appendix 8). Dispersion in Δ was modest: the SD was 3.2%, the IQR 4.2 percentage points and the 10th–90th percentiles −3.9% to +4.0%. Variation was slightly larger in practices with higher morbidity.

Overall redistribution under the morbidity-based formula was limited. The MAPC was 2.5% and the implied reallocation share was 1.26% (around 419 000 weighted patients), meaning that approximately one in eighty weighted patients would move across practices if national totals were held constant. Just over half of all patients (51.6%) were in practices with changes within ±2% and 86.7% within ±5%. A total of 299 practices (6.7%) gained at least 5% and 290 (6.5%) lost at least 5% and only 34 (0.8%) changed by 10% or more. Gains of at least 5% were most frequent in Q1 and Q2 and losses concentrated in Q3 to Q5 (table 3).

**Table 3:** Distribution of NHS practices by deprivation quintile and magnitude of change (Δ) under morbidity-inclusive weights

Change (Δ)	Q1	Q2	Q3	Q4	Q5	Total
Gainer >5%	66	72	63	36	62	299
Loser >5%	50	50	70	72	48	290
Small change <5%	778	767	759	773	774	3851
Total	894	889	892	881	884	4440

NHS, National Health Service.

We examined determinants of redistribution using patient-weighted linear regressions for the continuous outcome and linear probability models for crossing the ±5% threshold, with health authority fixed effects and standard errors clustered by health authority.

Higher morbidity strongly predicted upward movement. A 0.10 increase in QOF conditions per patient was associated with a +4.6-point rise in Δ, a 17.7-point higher probability of a≥5% gain and a 15.0-point lower probability of a ≥5% loss.

Older population profile predicted downward movement. A 10 percentage-point increase in the share aged 65 years and over was linked to a −9.0-point change in Δ, a −36.0-point lower probability of a≥5% gain and a 27.7-point higher probability of a≥5% loss.

Practice size had only a very small association with Δ.

Deprivation effects remained large after adjustment. Relative to IMD quintile 1, adjusted mean Δ was −1.30, −3.49, −5.59 and −7.15 points in quintiles 2 to 5 (all p<0.001). In the threshold models, practices in Q5 had an approximately 29-point lower probability of a ≥5% gain and a 20-point higher probability of a ≥5% loss.

Although the morbidity-based formula reallocates modestly toward practices with higher morbidity burden, practices in more deprived areas still lose relative share after accounting for recorded morbidity, age structure, practice size and region. This mirrors the patient-level fixed-effects results in IMRD and suggests that recorded morbidity does not fully capture the additional workload associated with socioeconomic disadvantage.

## Discussion

This study assessed how well a demographic-only capitation model reflects morbidity burden and how expected workload changes under a morbidity-inclusive alternative. Across complementary patient-level and practice-level analyses, morbidity emerged as a stronger predictor of annual primary care workload than demographic indicators alone. When applied nationally, morbidity-based weights led to modest redistribution, generally increasing expected workload for practices with higher morbidity and reducing it for those with older age profiles. However, socioeconomic differences persisted: even after adjusting for morbidity, age, list size and region, practices in more deprived quintiles continued to show lower residual weights.

At patient level, inclusion of QOF morbidity attenuated but did not eliminate the association between deprivation and workload, indicating that recorded long-term conditions capture only part of the additional workload associated with socioeconomic disadvantage. Morbidity adjustment therefore narrows, but does not remove, socioeconomic differences in predicted workload.

At practice level, deprived practices continued to show lower residual weights once morbidity and age structure were accounted for. If deprivation-related workload were fully captured by recorded morbidity, these residual differences would be expected to narrow substantially. Their persistence suggests that recorded morbidity does not fully capture the additional workload associated with socioeconomic disadvantage. The observed gradient therefore likely reflects undermeasurement of need rather than over-allocation under the demographic model.

Our findings align with previous research showing that capitation-based allocations correspond imperfectly with morbidity burden. Kontopantelis *et al* reported substantial geographic variation in chronic disease prevalence not reflected in funding levels.^1^ We extend this work by quantifying how demographic models, which rely heavily on age and sex, under-represent earlier morbidity in more deprived populations and by showing that morbidity adjustment only partially corrects this imbalance. The persistence of negative residuals in deprived practices echoes longstanding concerns about the inverse care law,^21^ and is consistent with structural supply constraints operating alongside under-recorded morbidity in deprived settings.

Recent person-based studies, such as the work by Anselmi *et al*, demonstrate that more detailed morbidity indicators and inclusion of ethnicity and deprivation account for more workload variation and direct more resources towards deprived practices.^4^ Our analyses complement this by showing that morbidity-based specifications reweight allocations in the expected direction but still leave a deprivation-related gap unaccounted for by recorded morbidity alone.

The redistribution patterns observed here reflect the design of demographic and morbidity-informed approaches. Demographic-only specifications weight age strongly, favouring practices with older populations, whereas morbidity-inclusive models shift weight towards recorded disease burden, which accumulates earlier in more deprived groups. Theories of ageing and morbidity trends help interpret these findings: evidence for the expansion of morbidity, with chronic illness rising faster than mortality declines, implies that models relying primarily on demographic proxies may become increasingly misaligned with the distribution of primary care workload.^22^

### Strengths and limitations

Strengths of this study include the use of national administrative data alongside a patient-level electronic health record dataset. The practice-level analysis examined variation in the NHS weighted practice index, while patient-level models estimated primary care workload using recorded consultation time weighted by staff role. Fixed-effects regression controlled for systematic differences between practices, enabling associations between patient characteristics and workload to be estimated independently of differences in staffing, organisation or coding. Predicted workloads used in the national simulations therefore reflect variation in patient characteristics rather than differences in service supply.

However, several limitations should be noted. Morbidity was measured using a restricted set of 17 recorded long-term conditions and may not capture the full range of clinical need or complexity contributing to workload. In addition, some elements of the Carr-Hill formula could not be replicated directly in the practice-level analysis and were approximated using available proxies. While this may introduce residual specification error, the relative contribution of age, deprivation and geography was stable across model specifications.

Under-recording of long-term conditions, particularly in more deprived settings, may contribute to conservative estimates of morbidity-related need. Some practice characteristics, such as ethnicity and workforce composition, were unavailable, and analyses were restricted to 2015–2017 to ensure temporal consistency across patient-level and practice-level datasets. More recent IMRD data are not available for re-analysis due to licensing constraints. However, the study examines the structural relationship between demographic weighting and morbidity within the NHS capitation framework, and the key features of this formula have not materially changed since the study period. The findings should therefore be interpreted as relating to the structure of the weighting model rather than to time-specific consultation volumes.

### Conclusions

Incorporating recorded morbidity into primary care workload modelling improves prediction of workload relative to demographic indicators alone and leads to modest but systematic redistribution of expected funding. Morbidity-informed specifications shift weight towards practices serving populations with higher recorded disease burden, whereas demographic-only models favour older population structures. However, adjusting for recorded morbidity does not eliminate socioeconomic differences in predicted workload.

More comprehensive measurement of morbidity and related drivers of workload could improve alignment between predicted workload and resource allocation if weighting approaches were revised.

## Supplementary material

10.1136/bmjopen-2025-114094 online supplemental file 1

## Data Availability

Data may be obtained from a third party and are not publicly available. All data relevant to the study are included in the article or uploaded as supplementary information.
