# Supplementary material for: Implications of incorporating morbidity into primary care workload models for NHS funding allocations: a retrospective observational study in England
Source: BMJ Open. 2026 Jun 25;16(6):e114094. doi: 10.1136/bmjopen-2025-114094 (PMC13311573; doi:10.1136/bmjopen-2025-114094)
Supplement: online supplemental file 1 [file bmjopen-16-6-s001.docx]

## Appendix 1A: Final model specification (Analysis 1)

**Outcome.** Practice index = (NHS weighted patients) / (registered patients).

**Study sample.** 4,440 GMS practices (England, calendar year 2016). Exclusions: part-year GMS contracts, atypical practices (large list size changes/closures), missing QOF or implausible funding.

**Final estimator.** Linear regression with health-authority fixed effects, cluster-robust SEs at HA level, unweighted (primary); list-size analytical weights used in sensitivity analysis.

**Covariates.**

Rurality: Urban vs Rural (reference = Rural)

Age structure: % aged ≥65 years (continuous, per pp)

Deprivation: IMD (2015) quintiles (reference = Q1 least deprived)

Morbidity: practice morbidity index = (sum of patients on each of 17 QOF registers) / list size

Market Forces Factor (MFF): continuous

Health authority dummies (reference = London)

**Model equation.**

$$\text{Index}_{i}\text{ }=\text{ }\beta_{0}+\beta_{1}\text{Urban}_{i}+\beta_{2}\%{65+}_{i}+\sum_{q=2}^{5} \beta_{3q}\text{ }\text{IMD}_{iq}+\beta_{4}\text{Morbidity}_{i}+\beta_{5}\text{MFF}_{i}+\sum_{h\neq\text{London}} \gamma_{h}\text{ }1(\text{HA}_{i}=h)+\varepsilon_{i},$$

with standard errors clustered by health authority.

**Stata implementation (primary).**

- Base categories

fvset base 1 imd2015 // IMD Q1 as reference

fvset base "LONDON" ha //

- Final FE-by-HA model (OLS with HA dummies), cluster SEs by HA

regress practice_index i.urban c.pct65 i.imd2015 c.morbidity c.MFF i.ha, vce(cluster ha)

- Regional contrasts vs London (predicted means by HA, holding covariates at observed values)

margins ha

margins, pwcompare(effects) baseout // pairwise vs base (London)

**Goodness of fit and variance decomposition.**

Overall fit: $R^{2}=0.769$.

Partial $R^{2}$(squared partial correlations) computed via Wald tests with cluster-robust VCE; normalised to sum to 100% across predictors (see Appendix 8).

**Diagnostics (summarised).**

LM test for RE: chibar²(1)=12,178.6, p<0.001 → between-HA variance present.

ICC (RE model): 0.27 (95% CI 0.15–0.44).

Robust Hausman (Sargan-Hansen): χ²(7)=109.5, p<0.001 → RE inconsistent → adopt HA fixed effects.

**Sensitivity analyses.**

- Random-effects sensitivity

xtset ha

xtreg practice_index i.urban c.pct65 i.imd2015 c.morbidity c.MFF, re vce(cluster ha)

- List-size weighting sensitivity (HA FE via dummies)

regress practice_index i.urban c.pct65 i.imd2015 c.morbidity c.MFF i.ha [aw=list_size], vce(cluster ha)

Key coefficients and HA contrasts remained directionally and statistically consistent; magnitudes stayed within the original 95% CIs.

## Appendix 1B: Model specifications (Analysis 2)

Two fixed-effects linear regression models were fitted at the patient level using IMRD-UK data to predict total annual patient workload (minutes per year, weighted by staff role and relative salary).

Models were estimated with analytical weights equal to person-years, and practice identifiers were absorbed to control for unobserved, time-invariant practice characteristics (areg …, absorb(practice_id) in Stata).

Robust standard errors were clustered at the practice level.

| Model | Dependent variable | Independent variables | Notes |
| --- | --- | --- | --- |
| Demographic-only | Annual patient workload (minutes) | Age-sex group (interaction), deprivation quintile, new-patient status | Represents a “baseline” specification analogous to a demography-only funding model |
| Morbidity-inclusive | Annual patient workload (minutes) | All variables from demographic-only model + 17 QOF long-term condition indicators + recent-diagnosis flag | Captures additional workload associated with diagnosed morbidity |

**Missing data:**

See Appendix 2.

**Calibration:**

See Appendix 9.

## Appendix 2: imputation specification for missing deprivation values

Missing deprivation quintile values were imputed in Stata using the mi impute ologit command. The imputation model treated deprivation quintile as an ordered categorical variable and included patient age band, sex, new patient status, morbidity count, practice identifier and clinical workload (the outcome variable). Approximately 18% of records had missing deprivation values; deprivation was the only variable with missing data, and all other covariates were complete. Missingness was assumed to be at random, conditional on the included covariates. Twenty imputations were generated using chained equations, and estimates were combined across datasets using Rubin’s rules (mi estimate). Diagnostic checks indicated plausible and stable imputations.

## Appendix 3: Practice inclusion and characteristics, NHS England dataset

| Inclusion and exclusion criteria | Number of practices excluded | Remaining practices |
| --- | --- | --- |
| Total practices in England 2016/17 | - | 7,763 |
| Excluded: non-GMS practices and practices with part-year GMS contracts | 2,980 | 4,783 |
| Excluded: atypical practices (large registration fluctuations or part-year data due to closure) | 301 | 4,482 |
| Excluded: missing QOF data or implausible funding per patient (<£10) | 11 | 4,440 |
| Final analytic sample | - | 4,440 |

| Health Authority | No. of practices | no of patients | Morbidity-to-practice index ratio | Morb-idity^[[1]](#footnote-1)^ | Deprivation score | % ≥65 |
| --- | --- | --- | --- | --- | --- | --- |
| LONDON | 617 | 3,789,055 | 4.25 | 0.40 | 24.3 | 11.0 |
| CENTRAL MIDLANDS | 395 | 3,370,762 | 5.43 | 0.53 | 16.7 | 17.2 |
| EAST ENGLAND | 385 | 3,159,236 | 5.43 | 0.54 | 17.9 | 19.6 |
| NORTH MIDLANDS | 267 | 2,122,780 | 5.63 | 0.58 | 21.6 | 19.1 |
| WEST MIDLANDS | 521 | 3,667,361 | 5.31 | 0.53 | 26.4 | 16.2 |
| CHESHIRE & MERSEYSIDE | 219 | 1,489,583 | 5.53 | 0.60 | 26.8 | 18.1 |
| CUMBRIA & NE | 311 | 2,262,357 | 5.57 | 0.61 | 25.9 | 19.1 |
| GTR MANCHESTER | 286 | 1,776,428 | 5.10 | 0.53 | 30.2 | 14.3 |
| LANCASHIRE | 148 | 951,706 | 5.71 | 0.59 | 23.4 | 18.5 |
| YORKS & HUMBER | 317 | 2,449,471 | 5.52 | 0.57 | 23.0 | 19.0 |
| SOUTH CENTRAL | 273 | 2,446,737 | 5.19 | 0.51 | 12.1 | 18.0 |
| SOUTH EAST | 392 | 3,241,087 | 5.40 | 0.53 | 16.6 | 19.0 |
| SOUTH WEST | 132 | 984,363 | 5.79 | 0.61 | 20.2 | 23.0 |
| SOUTH COAST | 177 | 1,606,801 | 5.62 | 0.56 | 15.4 | 20.6 |
|  |  |  |  |  |  |  |
| **Total** | **4,440** | **33,317,727** | **5.27** | **0.53** | **21.2** | **17.2** |

## Appendix 4: Partial R2 and relative contribution of predictors

The relative contribution of predictors was summarised using partial R² values derived from cluster-robust Wald statistics. For single-parameter terms, partial R² was computed from the squared robust t statistic:

Let $df_{2}$be the residual degrees of freedom from the model estimated with cluster-robust variance.

$$\text{partial }R_{j}^{2}\text{ }=\frac{t_{j}^{2}}{t_{j}^{2}+df_{2}}$$

where $df_{2}$is the residual degrees of freedom from the regression model estimated with cluster-robust variance.

For multi-parameter sets (e.g. deprivation quintiles, rurality and health authority fixed effects), partial R² was computed from the robust Wald *F* statistic:

$$\text{partial }R_{S}^{2}\text{ }=\frac{q\text{ }F_{S}}{q\text{ }F_{S}+df_{2}}$$

where $q$is the number of parameters jointly tested. Health authority fixed effects were treated as a categorical factor with $q=$(number of health authorities - 1) degrees of freedom.

Partial R² values were calculated for each single variable and multi-parameter set, then normalised to sum to 100%, providing approximate percentage shares of explained variance attributable to each factor. Calculations were implemented in Stata using robust t and F statistics from the fitted model (regress ..., vce(cluster ha)), with test and testparm commands used for multi-parameter sets.

**Table A4.1: Partial R2 values and relative contribution of predictors to**

**explained variance in the practice index (Analysis 1)**

| **Predictor** | **Partial R^2^** | **Share of explained variance (%)** |
| --- | --- | --- |
| % Aged 65+ | 0.995 | 19% |
| Morbidity index | 0.557 | 11% |
| Market Forces Factor | 0.774 | 15% |
| Rurality (urban/rural) | 0.961 | 18% |
| Deprivation (IMD quintiles) | 0.988 | 19% |
| Health authority (set) | 1.000 | 19% |

## Appendix 5: Coefficients of QOF regression model

| Sex |  |  |  |  |  |
| --- | --- | --- | --- | --- | --- |
| Female | -2.3 | 0.2 | 0.0 | -2.7 | -2.0 |
|  |  |  |  |  |  |
| Age group |  |  |  |  |  |
| <1 year | 27.2 | 0.7 | 0.0 | 25.8 | 28.5 |
| 01-04 years | ref |  |  |  |  |
| 05-09 years | -17.1 | 0.3 | 0.0 | -17.6 | -16.5 |
| 10-14 years | -17.0 | 0.3 | 0.0 | -17.6 | -16.5 |
| 15-19 years | -16.3 | 0.3 | 0.0 | -16.9 | -15.6 |
| 20-24 years | -14.0 | 0.4 | 0.0 | -14.7 | -13.3 |
| 25-29 years | -12.3 | 0.3 | 0.0 | -13.0 | -11.6 |
| 30-34 years | -9.2 | 0.3 | 0.0 | -9.9 | -8.6 |
| 35-39 years | -5.9 | 0.4 | 0.0 | -6.7 | -5.2 |
| 40-44 years | -3.4 | 0.4 | 0.0 | -4.1 | -2.6 |
| 45-49 years | -1.3 | 0.4 | 0.0 | -2.0 | -0.6 |
| 50-54 years | 0.0 | 0.4 | 0.9 | -0.7 | 0.8 |
| 55-59 years | 2.5 | 0.4 | 0.0 | 1.7 | 3.2 |
| 60-64 years | 5.2 | 0.4 | 0.0 | 4.5 | 6.0 |
| 65-69 years | 6.2 | 0.4 | 0.0 | 5.5 | 7.0 |
| 70-74 years | 10.4 | 0.5 | 0.0 | 9.6 | 11.3 |
| 75-79 years | 18.0 | 0.6 | 0.0 | 16.9 | 19.1 |
| 80-84 years | 25.8 | 0.7 | 0.0 | 24.4 | 27.1 |
| 85+ years | 33.0 | 0.9 | 0.0 | 31.3 | 34.8 |
|  |  |  |  |  |  |
| Sex/age group interaction |  |  |  |  |  |
| female#<1 year | -3.7 | 0.6 | 0.0 | -5.0 | -2.5 |
| female#01-04 years | ref |  |  |  |  |
| female#05-09 years | 3.5 | 0.2 | 0.0 | 3.0 | 3.9 |
| female#10-14 years | 4.5 | 0.2 | 0.0 | 4.0 | 4.9 |
| female#15-19 years | 19.9 | 0.3 | 0.0 | 19.2 | 20.6 |
| female#20-24 years | 23.0 | 0.5 | 0.0 | 22.1 | 24.0 |
| female#25-29 years | 23.3 | 0.5 | 0.0 | 22.4 | 24.2 |
| female#30-34 years | 25.5 | 0.4 | 0.0 | 24.7 | 26.3 |
| female#35-39 years | 23.5 | 0.4 | 0.0 | 22.8 | 24.3 |
| female#40-44 years | 21.3 | 0.4 | 0.0 | 20.6 | 22.0 |
| female#45-49 years | 21.3 | 0.4 | 0.0 | 20.6 | 22.0 |
| female#50-54 years | 21.8 | 0.4 | 0.0 | 21.0 | 22.5 |
| female#55-59 years | 17.9 | 0.4 | 0.0 | 17.1 | 18.6 |
| female#60-64 years | 15.2 | 0.4 | 0.0 | 14.4 | 16.0 |
| female#65-69 years | 15.7 | 0.4 | 0.0 | 14.9 | 16.4 |
| female#70-74 years | 17.5 | 0.5 | 0.0 | 16.6 | 18.4 |
| female#75-79 years | 18.8 | 0.6 | 0.0 | 17.7 | 20.0 |
| female#80-84 years | 15.9 | 0.7 | 0.0 | 14.5 | 17.4 |
| female#85+ years | 6.9 | 0.8 | 0.0 | 5.4 | 8.4 |
|  |  |  |  |  |  |
| Deprivation quintile |  |  |  |  |  |
| Least deprived Q1 | ref |  |  |  |  |
| Q2 | 1.1 | 0.1 | 0.0 | 0.8 | 1.4 |
| Q3 | 3.0 | 0.2 | 0.0 | 2.6 | 3.3 |
| Q4 | 5.6 | 0.2 | 0.0 | 5.2 | 6.0 |
| Most deprived Q5 | 8.3 | 0.2 | 0.0 | 7.9 | 8.8 |
|  |  |  |  |  |  |
| new patient | 7.7 | 0.3 | 0.0 | 7.1 | 8.3 |
| Arrhythmia | 33.1 | 0.6 | 0.0 | 31.9 | 34.3 |
| Asthma | 19.7 | 0.4 | 0.0 | 18.9 | 20.5 |
| Cancer | 7.5 | 0.3 | 0.0 | 6.9 | 8.1 |
| Chronic kidney disease | 20.0 | 0.6 | 0.0 | 18.9 | 21.2 |
| Rheumatoid Arthritis | 59.3 | 1.2 | 0.0 | 57.0 | 61.7 |
| COPD | 46.3 | 0.7 | 0.0 | 45.0 | 47.7 |
| Ischaemic heart disease | 20.1 | 0.5 | 0.0 | 19.2 | 21.0 |
| Dementia | 19.5 | 1.3 | 0.0 | 16.9 | 22.0 |
| Depression | 27.0 | 0.4 | 0.0 | 26.1 | 27.9 |
| Diabetes | 42.7 | 0.6 | 0.0 | 41.6 | 43.8 |
| Epilepsy | 40.7 | 1.3 | 0.0 | 38.2 | 43.2 |
| Heart failure | 40.6 | 0.9 | 0.0 | 38.8 | 42.4 |
| Hypertension | 14.5 | 0.3 | 0.0 | 14.0 | 15.0 |
| Learning disability | 28.7 | 0.8 | 0.0 | 27.1 | 30.3 |
| Mental health disorders | 61.6 | 1.9 | 0.0 | 58.0 | 65.3 |
| PVD | 24.1 | 0.8 | 0.0 | 22.6 | 25.7 |
| Stroke/TIA | 24.6 | 0.5 | 0.0 | 23.6 | 25.5 |
| Recent diagnosis | 16.5 | 0.4 | 0.0 | 15.8 | 17.2 |
| Constant | 33.1 | 0.4 | 0.0 | 32.3 | 33.9 |

## Appendix 6: Robustness checks for practice fixed effects

### Overview

Practice fixed-effects ($\alpha_{j}$) were obtained from patient-level models of consultation workload using the areg command with practice absorbed as a fixed effect. These coefficients represent practice-level differences in workload after controlling for age, sex, deprivation, morbidity, and new-patient and recent-diagnosis indicators. To ensure that the deprivation gradient in fixed-effects was not an artefact of model specification or data handling, we carried out a series of robustness checks.

### Alternative summaries of fixed-effects

We summarised fixed-effects across deprivation quintiles using several approaches: (i) untrimmed means, (ii) 5th-95th percentile trimmed means, and (iii) patient-weighted medians. In all cases, the pattern remained consistent - mean fixed-effects were lowest (most negative) in more-deprived quintiles, indicating lower workload delivery relative to predicted need. Trimming reduced the influence of extreme values but did not alter the direction or ordering of the gradient.

### Sensitivity to small practices

To check that results were not driven by outliers among very small practices, we re-estimated deprivation summaries after excluding the smallest 10% of practices by list size. The deprivation gradient persisted with minimal change in magnitude.

### Handling of deprivation and missing data

Missing Townsend deprivation scores were imputed using multiple imputation with 20 iterations in the main analysis. As a sensitivity, we repeated the analysis using observed-only deprivation values, recalculating quintiles from the modal observed score per practice. With observed-only data, the gradient became more clearly monotonic, suggesting that the pattern seen with the imputed data was not caused by the imputation.

### Alternative outcome: consultation counts versus minutes

The main model used total consultation minutes per patient-year as the workload outcome. To assess robustness to outcome definition, we repeated the fixed-effects models using consultation counts (GP + nurse) as the dependent variable. The deprivation ordering of fixed-effects remained unchanged, indicating that the gradient does not depend on the workload measure.

### Model specification and estimation

We compared fixed-effects and random-effects models using Hausman tests, which supported fixed-effects due to correlation between practice effects and observed covariates. The results were robust to clustering at the practice versus health authority level and to using either exposure weights (patient-years) or equal weights. Adding quadratic terms for age structure and morbidity did not materially change the deprivation gradient.

### Summary

Across all checks - alternative summaries, exclusion of small practices, observed-only deprivation, alternative workload outcome, and alternative model specifications - the deprivation gradient in practice fixed-effects remained robust in sign and direction. This suggests that residual shortfalls in deprived practices are unlikely to arise from model choice or measurement artefacts and more plausibly reflect under-measured need or capacity constraints.

## Appendix 7

The practice index is the ratio of weighted to registered patients, rescaled to a mean of 100. Under the demographic-only model, mean indices ranged from 98.0 in Q1 to 101.5 in Q5 (Q5-Q1 difference 3.5 points, 3.6%). Under the morbidity-inclusive model, the range widened from 97.3 to 103.1 (difference 5.8 points, 5.9%). Thus, including morbidity modestly steepened the deprivation pattern at practice level, reflecting slightly higher predicted workload in deprived areas once morbidity was accounted for.

The patient-weighted correlation between demographic and morbidity-inclusive indices was high (r 0.86). Dispersion increased slightly: the weighted SD rose from 6.5 to 7.4, and the 90-10 percentile spread from 15.2 to 18.7. Morbidity adjustment therefore increased variation in expected workload and modestly reranked practices.

Redistribution effects were small but systematic. The mean absolute proportional change (MAPC) was 0.025 (2.5%), with an interquartile range of 0.011 to 0.036. The 95th percentile change was 6.1%, and the maximum was 22.7%, indicating that large shifts were uncommon.

Gains and losses by deprivation quintile are shown in Table A7.1. Unadjusted counts suggest gains were more common in the most deprived quintiles (Q4-Q5) and losses more frequent in the least deprived (Q1-Q2), indicating redistribution toward higher-need populations. These patterns differ from those observed in the full national simulation (Analysis 3), reflecting differences in sample composition and adjustment.

**Table A7.1 Distribution of IMRD practices by deprivation quintile and**

**magnitude of change (Δ) under morbidity-inclusive weights**

| Change (Δ) | Q1 | Q2 | Q3 | Q4 | Q5 | Total |
| --- | --- | --- | --- | --- | --- | --- |
| Gain > 5% | 13 | 6 | 11 | 21 | 23 | 74 |
| Loss > 5% | 21 | 8 | 12 | 7 | 4 | 52 |
| Small change < 5% | 126 | 74 | 114 | 105 | 82 | 501 |
| Total | 160 | 88 | 137 | 133 | 109 | 627 |

Regression analyses showed that morbidity and age structure largely drove these shifts. Each additional 0.10 conditions per patient was associated with a +3.6 percentage point increase in Δ. A 10 percentage point increase in the population aged ≥65 reduced Δ by about 6 percentage points. These effects influenced redistribution probabilities: higher morbidity increased chances of a ≥5% gain and reduced chances of a ≥5% loss; a larger older population had the opposite effect.

After adjusting for morbidity, age structure and region, deprivation effects reversed relative to the unadjusted counts: mean Δ in Q5 was 2.4 percentage points lower than in Q1, and practices in Q5 were about 20 points more likely to experience a ≥5% loss. This indicates that the apparent gains in deprived quintiles are driven by higher morbidity and younger age profiles; once these are held constant, deprived practices remain relatively worse off, consistent with under-measurement of need.

## Appendix 8: Assessment of the symmetry of the distribution of Δ

To evaluate whether the distribution of proportional changes (Δ) was approximately symmetric around zero, three complementary checks were performed:

1. **Visual inspection** of histograms and kernel density plots across all practices.
2. **Comparison of mean and median** values, where approximate equality indicates near-symmetry.
3. **Calculation of the sample skewness statistic**,

$$\text{skewness}=\frac{E\{(\Delta-\Delta)^{3}\}}{s^{3}},$$

where $\Delta$is the mean and $s$is the standard deviation. Skewness values close to zero indicate symmetry, while positive or negative values indicate right or left skew, respectively.

As an additional check, the median of Δ was formally tested against zero using both the sign test and a binomial test.

Across 4,440 practices, the kernel density plot (Figure A8.1) showed a distribution centred close to zero with minimal asymmetry. The mean (-0.0005) and median (-0.0010) were nearly identical, with a skewness of 0.29 (Table A8.1). Formal tests provided no evidence that the median differed from zero (sign test *p* = 0.10; binomial test *p* = 0.10). These results indicate that the distribution of Δ was approximately symmetric, supporting the use of both mean/SD and median/IQR summaries.

Table A8.1: Summary statistics and symmetry tests for proportional changes (Δ)

| **Statistic** | **Value** |
| --- | --- |
| Mean | -0.0005 |
| Median | -0.0010 |
| SD | 0.0343 |
| IQR | 0.0442 |
| Skewness | 0.29 |
| Sign test *p*-value | 0.10 |
| Binomial test *p*-value | 0.10 |


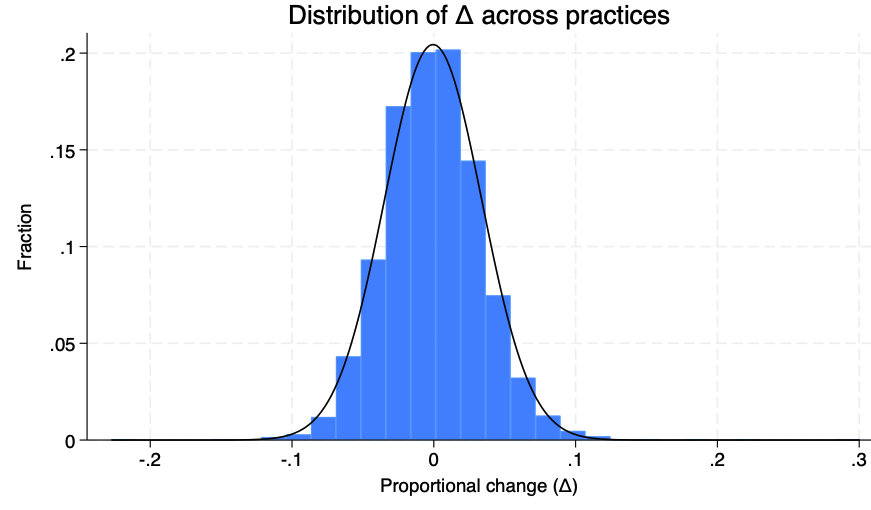


Figure A8.1: Distribution of Δ across practices

## Appendix 9: Calibration

Calibration was assessed by regressing observed on expected workload at the practice level:

$$\text{Observed}_{p}=\alpha+\beta\cdot\text{Expected}_{p}$$

We report the calibration slope (β), R², and calibration-in-the-large (CITL). Confidence intervals were obtained using robust standard errors. Slopes close to one indicate good proportional calibration (β > 1 = under-prediction at high workloads; β < 1 = over-prediction).

For reference, pmcalplot also reports CITL as the mean difference (Observed - Expected). Identical pmcalplot CITL values across models reflect their similar overall predicted means, while model differences are primarily captured by the slope.


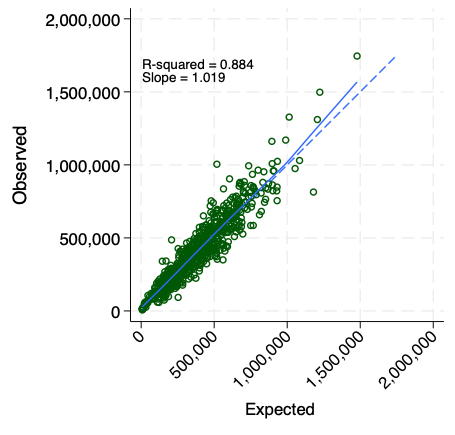
Figure A9.1 shows the calibration plots for both models.


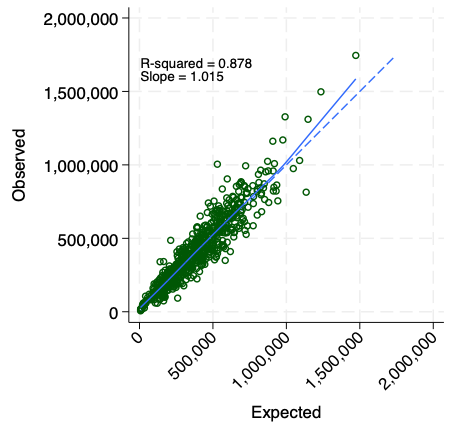


**Figure A9.1: Calibration plots of demographic-only model (left) and morbidity-inclusive model (right)**

Calibration at practice level was high. Regressing observed on expected workload produced slopes near one: demographic-only β 1.015 (R^2^ 0.878) and morbidity-inclusive β 1.019 (R^2^ 0.884), indicating mild under-prediction at higher workloads. Calibration-in-the-large was almost identical across models (about 23,000 unweighted). Intercepts differed modestly (17,136 vs 15,670).

## Appendix 10

We summarised Δ using the median, interquartile range, 10th and 90th percentiles, the mean absolute proportional change (MAPC) and the Wald share:

$$\frac{1}{2}\frac{\sum\left| W_{morbidity, p}- W_{demographic, p} \right|}{\sum_{p} T_{p}},$$

where $W_{\text{demographic},p}$ and $W_{\text{morbidity},p}$ denote the total weighted patients for practice $p$under each model, and $T_{p}$is the practice list size.

1. QOF morbidity index = total QOF disease register counts ÷ registered list size (average number of QOF-recorded chronic conditions per patient). Higher values indicate greater morbidity burden. [↑](#footnote-ref-1)
