# Supplementary material for: Implications of incorporating morbidity into primary care workload models for NHS funding allocations: a retrospective observational study in England
Source: BMJ Open. 2026 Jun 25;16(6):e114094. doi: 10.1136/bmjopen-2025-114094 (PMC13311573; doi:10.1136/bmjopen-2025-114094)
Supplement: Supplementary file 2 [file bmjopen-2025-114094.reviewer_comments.pdf]

PEER REVIEW HISTORY

BMJ Open publishes all reviews undertaken for accepted manuscripts. Reviewers are asked to complete a checklist review form and are provided with free text boxes to elaborate on their assessment. These free text comments are reproduced below.

ARTICLE DETAILS

Title (Provisional)

Implications of incorporating morbidity into primary care workload models for NHS funding allocations: a retrospective observational study in England

Authors

de Dumast, Lyvia; Moore, Patrick; Snell, Kym; Marshall, Tom

VERSION 1 - REVIEW

**Reviewer**	**1**
**Name**	**Kadel, Rajendra**
**Affiliation**	**Public Health Wales, Policy and International Health**
**Date**	**09-Jan-2026**
**COI**	**None**

A rigorous, policy-relevant observational study using high-quality data and appropriate methods. The paper is suitable for BMJ Open pending minor–moderate revisions to improve interpretation and clarity.

Major comments:

1. Interpretation of “need-only” workload

Setting practice fixed effects to zero embeds a normative assumption about need vs supply. This should be stated more explicitly and briefly justified.

2. Deprivation effects

The persistence (and widening) of deprivation gradients after morbidity adjustment is a key finding and needs clearer theoretical interpretation.

3. Morbidity specification

Reliance on QOF conditions likely underestimates need in deprived populations; conclusions should be framed as a lower bound on morbidity-related redistribution.

Minor comments

- Clarify deprivation quintile ordering (eg. Q1 least, Q5 most deprived) early and consistently.

- Improve consistency in terminology for comparator models (baseline / demographic-only / current NHS formula).

- Briefly justify workload staff-weight assumptions in the main text.

- Add a short plain-language explanation of the redistribution (Wald share) metric.

- Explicitly acknowledge limited contemporary relevance given post-2017 changes in primary care.

**Reviewer**	**2**
**Name**	**Smith, Samantha**
**Affiliation**	**Economic and Social Research Institute, Dublin**
**Date**	**15-Jan-2026**
**COI**	**None**

General comments:

Quite clearly a considerable amount of work has gone into the analysis in this paper and could make an important contribution to the field.

However, the paper itself needs a good deal more work to provide context and justification for the analysis, to explain the data and the analysis more clearly, to present results more clearly (e.g., most of the tables and figures are untitled) and to discuss the findings in more depth. The main focus in this review is on the need for better communication of the content because without this it is hard to comment on the analysis and findings.

Detailed comments:

1. Introduction

The introduction reads more like a methods section than an introduction. Two out of the three paragraphs outline the proposed analysis. The section needs more background information to contextualise and motivate the analysis in the paper.

For example, we need:

A general introduction to primary care and general practice structures and funding in England (e.g., what is the Global Sum for, how much is it, what are the other funding components, etc.).

Background literature on weighed capitation formulas, concerns about existing mechanisms (and supporting evidence), more detailed justification for this analysis.

2. Methods

Setting: A description of the Setting would be useful here: e.g., population, number of GP practices, average number of visits per year, average funding allocations and the degree of variation in allocations, average practice size etc.

Data:

- The description of the data and methods assumes the reader has in-depth local knowledge of the datasets, what all of the variables mean and where they come from. For example, it is not clear until much later in the paper what metric is used to measure workload.

- The description of the NHS practice-level data would make much more sense if better background information on general practice in England was provided in the introduction (e.g., 'general medical services (GMS) practices', health authorities). Even at that, some of the variables need further explanation for non-UK readers unfamiliar with the setting: quality and outcomes framework registers, market forces factor index. In particular, it is really difficult to understand how the QOF variable is used as a morbidity indicator without further explanation.

- IMRD dataset (IMRD acronym not explained). Townsend measure of deprivation is explained later in the paper but should be clearly outlined here (and referenced). Similarly, Read codes need to be explained.

- Terminology: the descriptions of need, demand and supply are interesting - it could be argued that observed workload reflects both demand and supply-side factors. I may need GP care but choose not to attend (so need is there but demand is zero), or I may attend GP care but not actually need anything (but the GP's time has already been taken up in determining that fact) (so demand is there but need is zero). Perhaps the wording needs to be adjusted here to acknowledge that the definition of these terms is more complicated - e.g., "For the purposes of this paper we define need as...."?

Analyses:

- This section needs to be revised in light of clearer explanation of the datasets and variables above.

e.g., Analysis 1: 'A morbidity index was calculated as the sum of all QOF disease-register counts....." - means very little unless you are familiar with the QOF variable.

- Revision is also needed to explain the proposed analyses more carefully. Some of the proposed analyses only become clear once the results are outlined. A diagram/chart could help to summarise the approach used.

e.g., Analysis 1: the purpose of the model becomes more clear in the results section. It would be helpful to spell out the purpose of the model in the methods section.

- Modelling strategy, Analysis 1: is there a problem of simultaneity here with the same variables appearing on both sides of the equation? Implications for choice of model?

i.e., Dependent variable: the practice index calculated as ratio of weighted to registered patient numbers, where it looks like the weighted numbers are calculated using the Carr-Hill formula by controlling for rural/urban, deprivation, market forces factor (and age and sex?); while the independent variables include a similar list: rural/urban, age, deprivation, market forces factor, and morbidity.

Results:

- Most of the figures and tables have no Figure/Table Number, No Title?

- Tables of regression results - sample size, model diagnostics, sources?

- Table of basic descriptives on the variables in each dataset would be helpful.

Results and discussion:

- Interpreting the results, without more contextual information, is difficult for the reader. For example, the reader has little sense of how large the variations are in funding allocations across practices in existing resource allocations.

- The reference to other work in this area seems limited.

**Reviewer**	**3**
**Name**	**Kelson, Zoe**
**Affiliation**	**University of Exeter, Mathematics**
**Date**	**02-Feb-2026**
**COI**	**None**

This retrospective observational study aims to assess how well the current NHS weighting formula reflects morbidity related workload and to examine how a morbidity-inclusive alternative would redistribute resources at national scale.

Reviewer comments:

"following the RECORD guideline"

Thanks to the authors for providing a copy of the RECORD statement.

"using patient-level electronic health records and national practice-level administrative data... Primary care in England, including 4,440 general medical services practices and over 4 million patients from IMRD-UK electronic health records" [Abstract]

and

"Two datasets were used. • NHS England practice-level data (2016/17). These data cover all general medical services (GMS) practices. Variables included weighted and registered list size, rurality, the 2015 Index of Multiple Deprivation (IMD), Quality and Outcomes Framework (QOF) registers, the Market Forces Factor index (MFF) and health authority

identifiers. • IMRD UK patient-level data (2015). IMRD-UK contains anonymised EHRs from UK practices, covering over four million patients (2). It is representative of the UK population (3). Data were extracted using the DExtER tool (4)."

Can the authors please further comment on the completeness and coverage of data?

These datasets are now about a decade old. Can data be updated and re-analysed prior to publication?

"Missing deprivation (18%) was multiply imputed using ordinal logistic regression including all analysis variables and the outcome (14,15). Full imputation details are in Appendix 2"

Can the authors please provide more information on missing data for all variables, and consider conducting a sensitivity analysis on the approach taken for handling missing data in the analysis?

"Analyses comprised: (1) practice-level modelling of the current NHS weighted practice index; (2) patient-level fixed-effects regression of clinical workload; and (3) national simulations applying morbidity-based coefficients to all practices, contrasted with a demographic-only analytic model constructed for comparison." [Abstract]

A suitable approach has been followed.

"We controlled for the main elements of the formula using available demographic and morbidity proxies. Some components could not be replicated directly, but proxy measures were included wherever feasible. "

Can the authors please undertake sensitivity analyses to explore uncertainty of proxy measures?

"A multivariable linear regression model was fitted with the NHS practice index as the dependent variable. Covariates were rurality, percentage aged 65 and over, IMD quintile, morbidity burden and MFF. Health authority fixed effects were included and standard errors were clustered by health authority...

Partial R^2 values summarised each predictor's relative contribution using cluster-robust Wald statistics. Full details of model specification and diagnostics are provided in Appendix 1A and 1B."

and

"In Analysis 2, fixed-effects regression was used to separate patient need from practice-level supply factors...

Fixed-effects linear regressions were fitted using areg, absorbing practice identifiers. Analytical weights equalled person-years. Standard errors were clustered by practice and estimates combined using Rubin's rules (17). A Hausman test assessed the fixed-effects specification (18). Two models were estimated: (i) a demographic-only model (age-sex,

deprivation, new-patient status) and (ii) a morbidity-inclusive model adding QOF morbidity indicators...

As a robustness check, models were re-estimated using consultation counts as the outcome with fixed-effects negative binomial regression."

and

"We applied the patient-level coefficients from the fixed-effects model to each practice's demographic and morbidity profile to generate expected workload per registered patient...

Two sets of coefficients from Analysis 2 were used: (i) a demographic-only model and (ii) a morbidity-inclusive model including QOF indicators and a recentdiagnosis flag. Both models used fixed-effects linear regression with cluster-robust standard errors and analytical weights equal to person-years...

Binary outcomes were modelled using linear probability models with robust standard errors clustered by health authority."

Appropriate modelling methods have been applied by the authors, suitably reflecting the structure of the data and undertaking sensitivity analyses which help to demonstrate the robustness of the study findings.

The Results are reported consicely and accurately, with clear and informative supportive detail provided in the Tables and Figures.

"Morbidity measures were limited to 17 QOF conditions and may under-record need, particularly in deprived areas; important domains such as mental health complexity, multimorbidity patterns, chronic pain, substance misuse and frailty were not captured. • Variation in workload recording and missing contextual factors (e.g. workforce mix, service configuration) may introduce residual bias and data from 2015- 2017 may not reflect recent service changes" [Strengths and Limitations of this study]

and

"However, several limitations should be noted. Morbidity measures were restricted to 17 QOF conditions; some important contributors to workload including non-QOF morbidity, multimorbidity complexity and psychosocial need, were not included in the morbidity predictors themselves. Under-recording of long-term conditions, particularly in more deprived settings, may contribute to conservative estimates of morbidity-related need. Some practice characteristics, such as ethnicity and workforce composition, were unavailable and analyses reflect the period 2015-2017, preceding recent changes in consultation mode and service delivery. These limitations suggest that deprivation-related workload may be underestimated."

A discussion of the study limitations is provided by the authors.

VERSION 1 - AUTHOR RESPONSE

Reviewer 1

We thank Reviewer 1 for their thoughtful and constructive comments.

1. Normative assumption embedded in "need-only" prediction.

Response: We have clarified this in the Methods section. The manuscript now explicitly states that setting practice fixed effects to zero reflects the assumption that funding allocations should be based on patient-level need rather than historical or structural differences in service supply. This clarification has also been referenced briefly in the Discussion.

2. Interpretation of deprivation gradient requires further explanation.

Response: The Discussion has been expanded to clarify the persistence of deprivation differences after morbidity adjustment. We now distinguish clearly between patient-level and practice-level findings and discuss potential mechanisms, including under-recorded morbidity, psychosocial complexity and structural supply constraints.

3. QOF-based morbidity may underestimate need.

Response: We have strengthened the limitations section to note that QOF-based measures likely provide a conservative estimate of morbidity-related workload, particularly in deprived settings.

Minor comments

Response: We have standardised deprivation quintile terminology, clarified comparator descriptions, explained the redistribution metric more clearly and provided brief justification for staff-role weighting assumptions.

We have also clarified in the limitations section that although consultation patterns and workforce configuration have evolved since 2015–2017, the structural features of the capitation formula and its reliance on demographic proxies remain unchanged. The findings therefore continue to inform interpretation of the current allocation framework.

Reviewer 2

We thank Reviewer 2 for their detailed and constructive comments regarding contextual clarity and presentation.

1. Introduction lacks sufficient context about NHS funding.

Response: We have substantially revised the Introduction to strengthen contextual background and motivation. Specifically, we have expanded the description of primary care funding structures in England, clarifying the role of the Global Sum within overall general

practice income and its relationship to other funding streams. We have also added discussion of the broader literature on capitation and risk adjustment, including evidence on the limitations of demographic-only models, concerns regarding morbidity measurement and equity, and prior UK analyses of morbidity and spending variation. These additions more clearly situate the study within existing research and articulate the empirical gap addressed by examining the allocation implications of morbidity-inclusive workload modelling at national scale.

2. Dataset and method descriptions assume local knowledge.

Response: We have revised the Methods section to improve clarity for readers unfamiliar with the datasets and variables. IMRD is now expanded and described at first mention, including information on coverage and representativeness. We have clarified the meaning and construction of key variables, including the practice index (defined as the ratio of formula-adjusted to registered patients), the Market Forces Factor (MFF), rural-urban classification (as provided in the NHS dataset) and deprivation measures.

3. How QOF registers function as morbidity indicators is unclear.

Response: We have clarified how QOF disease registers function as measures of diagnosed morbidity and how they are operationalised in both the practice-level and patient-level analyses. Specifically, we now explain that QOF registers record counts of patients diagnosed with specified long-term conditions and that these counts are used as proxies for recorded morbidity burden. We also clarify how the practice-level morbidity index is constructed and how patient-level QOF conditions are incorporated in regression models.

4. A descriptive statistics table is needed.

Response: A new Table 1 summarising key demographic, deprivation and morbidity characteristics of both the NHS practice-level and IMRD patient-level datasets has been added to the Results section.

5. Workload measure is not clearly defined.

Response: We have clarified early in the Methods section that workload is measured as annual consultation time per patient, expressed in minutes and weighted by staff role to reflect relative resource intensity.

6. Clarify analytic sequence and purpose of models.

Response: The Overview of Analyses section has been revised to describe more clearly the purpose and relationship of the three analyses and how they build sequentially from describing the current NHS weighting structure to modelling patient-level workload and simulating national allocation changes.

7. Modelling strategy in Analysis 1 re possible problem of simultaneity with similar variables appearing on both sides of the equation

Response: Analysis 1 is intended as a descriptive decomposition rather than a structural estimation of the Carr-Hill formula. The dependent variable (the published NHS practice index) is constructed using demographic and area-based adjustments under the Carr-Hill framework. By regressing the index on observable demographic and morbidity variables, we assess the relative contribution of these components to variation in the index. The analysis does not attempt causal inference or estimation of funding parameters, and therefore simultaneity in the econometric sense is not at issue.

8. Clarify relationship between dependent variables and predictors.

Response: We have clarified the distinction between the published NHS practice index (used as the dependent variable in Analysis 1) and the demographic and morbidity variables included as explanatory proxies. The revised text makes explicit that Analysis 1 examines the relative contribution of observable components rather than reconstructing the Carr-Hill formula in full.

Reviewer 3

We thank Reviewer 3 for their constructive feedback.

1. Clarify data completeness and coverage.

Response: The Methods section now clarifies the coverage and representativeness of both datasets.

2. Provide more information on missing data.

Response: We clarify that deprivation was the only variable with missing data (18%). All other covariates were complete. The imputation model is fully specified in Appendix 2.

3. Consider sensitivity analysis and proxy limitations.

Response: We discuss limitations of QOF-based morbidity measures in the Discussion and report sensitivity analyses in the appendices.

4. Can data be updated and re-analysed prior to publication?

Response: The analyses were restricted to 2015–2017 to ensure temporal consistency across the patient-level and practice-level datasets. Additional post-2019 IMRD data are not available for re-analysis due to licensing constraints. However, the study examines the structural relationship between demographic weighting and morbidity within the NHS capitation framework, and the key features of this formula have not materially changed since the study period. We have clarified this point in the limitations section.

Summary

The revised manuscript addresses all comments raised by the Editor and reviewers. We are grateful for the constructive feedback, which has strengthened the clarity, transparency and interpretability of the study.

VERSION 2 - REVIEW

**Reviewer**	**1**
**Name**	**Kadel, Rajendra**
**Affiliation**	**Public Health Wales, Policy and International Health**
**Date**	**11-Mar-2026**
**COI**	

The results in the abstract section is too technical and with limited interpretation. This need to be revised to make reader friendly. Results section also need revision with reader friendly interpretation of the findings.

**Reviewer**	**3**
**Name**	**Kelson, Zoe**
**Affiliation**	**University of Exeter, Mathematics**
**Date**	**27-Mar-2026**
**COI**	

Many thanks to the authors for their responses and revisions, which have strengthened the reporting of this study.

VERSION 2 - AUTHOR RESPONSE

Response to Reviewer 1
Thank you for this helpful comment. We have revised the Abstract and Results sections to improve readability and provide clearer interpretation of the findings.
In the Abstract, the Results paragraph has been rewritten to reduce technical phrasing and clarify the comparison between the demographic-only and morbidity-inclusive models. Ambiguous wording has been replaced with clearer descriptions of the analyses and findings.
In the main Results section, we reviewed the text throughout to ensure that findings are presented in a reader-friendly manner while retaining the necessary statistical detail. In particular, we:

• simplified several sentences that previously used methodological or model-focused wording;
• added brief interpretive statements highlighting the substantive implications of the results (for example, the relatively small redistribution implied by morbidity-based weights and the persistent deprivation gradient); and
• removed software-specific language and simplified descriptions of diagnostic checks where these were not essential to understanding the findings.
